# Prevalence, indications, and preference of caesarean section deliveries among women attending primary health care units in Port-Said City, Egypt

**DOI:** 10.1186/s42506-026-00218-1

**Published:** 2026-05-11

**Authors:** Nesrine S. Farrag, Khaled S. Salama, Almaza A. Salim, Ateya M. Ibrahim, Nesreen F. Ibrahim

**Affiliations:** 1 Community Medicine Department, Faculty of Medicine, Port Said University, Port Said, Egypt; 2 Orthopedic Surgery Department, Faculty of Medicine, Port Said University, Port Said, Egypt; 3 Family Medicine Department, Faculty of Medicine, Port Said University, Port Said, Egypt; 4 Family and Community Health Nursing, Faculty of Nursing, Port Said University, Port Said, Egypt; 5 College of Nursing, Prince Sattam bin Abdulaziz University, Al-kharj, Saudi Arabia

**Keywords:** Caesarean Section, Deliveries, Indications of Caesarean Section, Preference of Caesarean Section, Prevalence of Caesarean Section, Primary Health Care Units

## Abstract

**Background:**

With the changing perception of Caesarean Section (CS) from a lifesaving to a routine procedure, non-medically indicated CS is increasing. This study assessed the prevalence, indications, and preference for CS among women attending primary health care (PHC) units in Port Said Governorate, Egypt.

**Methods:**

A cross-sectional study was conducted from December 2023 to February 2024 in six primary health care units across Port-Said Governorate, Egypt. Married women of childbearing age who had experienced at least one childbirth (primiparous and multiparous) were included. A total of 179 participants were recruited using multistage sampling, combining random selection of PHC units and consecutive sampling of eligible women. Data were collected using a validated structured questionnaire covering socio-demographics, obstetric history, last delivery experience, and preference for the next delivery. Statistical analysis was performed using SPSS v26, including univariate and multivariate logistic regression to identify factors associated with CS and delivery preference.

**Results:**

A total of 179 women participated in the study. Of these, 68% had CS for their last delivery. The most frequently reported reasons for previous CS were fear of labor (56%), prolonged labor (18%), and long distance to the hospital (15.6%). Regarding future delivery preferences, (61.5%) of women expressed a preference for CS. The leading reasons cited were less pain (81.8% ), the belief that CS is safer for the baby (57.3%), and the knowledge of the time of delivery (32.2%). Multivariate logistic regression identified independent predictors for each outcome. Women with higher income [AOR 10.0, 95% CI 2.7–36.9, < 0.001] and those whose doctor suggested CS [AOR 19.2, 95% CI 5.5–67.1, *p* < 0.001] were more likely to have had a CS in their last delivery. In contrast, preference for CS in the next delivery was independently associated with higher husband education [AOR 12.1, 95% CI 1.2–124.3, *p* = 0.036] and a history of previous CS [AOR 14.7, 95% CI 6.2–34.6, *p* < 0.001].

**Conclusion:**

Fear of labor and previous CS were the main drivers of past and preferred future CS deliveries, with non-medical factors outweighing medical indications. Findings highlight the need for educational interventions to support informed delivery choices.

**Supplementary Information:**

The online version contains supplementary material available at 10.1186/s42506-026-00218-1.

## Introduction

Caesarean Sections (CSs) can be lifesaving for both mother and baby when it is medically justified, and the method of choice in high-risk pregnancies [1], but when their proportion exceeds 10% of live births, there is no associated decline in maternal and neonatal mortality. Conversely, the utilization of unnecessary CS, particularly in settings characterized by resource constraints, has been associated with an increased risk of short- and long-term maternal and perinatal complications, in addition to a substantial economic burden on society [2]. The rising trend of CS overuse is a global concern due to its potential risks for both mothers and infants [3].

Non-clinical indications for CS, often termed Caesarean delivery on maternal request (CDMR), refer to elective CS performed in the absence of medical or obstetric necessity, that is, without clinical risks such as fetal distress, placenta previa, or other medical indications [4]. These non-clinical CSs account for a substantial proportion of global caesarean deliveries and are driven by factors such as women’s preferences, e.g., fear of labor, concerns about pelvic floor outcomes, fear of litigation, provider practices, and broader sociocultural influences rather than medical need [5, 6]. It has been estimated that up to one-third of the approximately 18.5 million CSs performed annually worldwide are conducted without a clear medical indication, related to rising CS rates globally [7].

The high CS rate warrants monitoring indications of all CSs in public and private facilities [6]. The World Health Organization (WHO) has issued guidance on non-clinical interventions designed to reduce unnecessary CSs, including supportive labor practices, second-opinion policies, and structured clinical audits. These recommendations recognize that complex factors at the levels of women, providers, and health systems are all related to rising CS rates and must be addressed simultaneously [8].

In Egypt, CS rates have reached alarmingly high levels. According to the 2021 Egypt Health Survey, the national proportion of CS deliveries was approximately 72% of all births, which constitutes more than three times the WHO-recommended upper threshold and among the highest rates globally. Such high rates highlight the need to investigate underlying causes beyond clinical necessity [9].

Although national data demonstrate exceptionally high CS rates in Egypt, there is considerable subnational variation in CS utilization across governorates, reflecting differences in healthcare infrastructure, provider practices, and sociocultural factors. Port Said governorate has reported (91.3%) higher-than**-**national-average CS rates, yet there is a paucity of published research examining the local determinants of delivery mode in this setting [10]. Specifically, limited evidence is available regarding women’s preferences, non-clinical drivers, and contextual health system factors influencing CS use at the governorate level. This lack of localized data represents a critical knowledge gap, underscoring the need for governorate-specific studies to inform targeted interventions aimed at optimizing CS use in alignment with national and WHO recommendations [8].

An initiative was adopted by the faculty of medicine in Port Said University to set a long-term plan for lowering CS rates in Port Said governorate. The plan includes, among other strategies, a research component that provides baseline data for understanding the problem and for further evaluation of this plan. The current study is one of the outcomes of this initiative. This study aims to assess the prevalence and indications of CS among women attending PHC units in Port-Said Governorate, identify the predictors of CS deliveries, and evaluate preferences and predictors of the future mode of delivery.

## Methods

### Study design and settings

A cross-sectional study was conducted in Port Said city, Egypt. The overall study period extended from December 2023 to June 2024; however, data collection from participants was carried out between December 2023 and February 2024. The study was carried out in six PHC units, with one PHC unit randomly selected from each administrative district of Port Said to ensure geographic representation.

### Study population and eligibility criteria

The study population comprised married women attending the selected PHC units for any health-related reason. Inclusion criteria were married women who had experienced at least one previous childbirth. Both primiparous women (one previous delivery) and multiparous women (two or more previous deliveries) were included, as these groups may differ in obstetric experience, risk perception, and preferences regarding mode of delivery, particularly elective CS on maternal request. Including both groups allowed a more comprehensive assessment of factors influencing delivery choices. Parity definitions were based on standard obstetric terminology [11]. Women who had never given birth or were unable to provide informed consent were excluded.

### Sample size

The sample size was calculated using Epidemiological Information Software (EPI INFO^™^) software v. 7.2.2.16, based on a reported CS prevalence of 91.3% in Port Said governorate from the Egypt Health Issues Survey 2021 [12]. The calculation assumed a 95% confidence level, 80% **s**tudy power, a 5% margin of error, and a design effect of 1.25 to account for the multistage sampling technique. The minimum required sample size was 153 participants. After adding 10% to compensate for potential non-response, the final estimated sample size was 167 women. Ultimately, data were collected from 179 participants, exceeding the minimum requirement.

### Sampling procedure

Multistage sampling was conducted. In the first stage, simple random sampling was performed to select one PHC from each district. In the second phase, participants were recruited using a convenience sampling method. This approach was adopted due to practical constraints for recruiting participants within busy primary healthcare settings and the absence of a complete sampling frame for all eligible women in the catchment areas. Women who were present in the waiting areas of the selected PHC units during clinic hours were approached consecutively by the researchers. All approached women were assessed for eligibility according to the predefined inclusion and exclusion criteria. Those who met the eligibility requirements were informed of the study’s objectives and procedures and were invited to participate. Data collection was undertaken by two researchers, each responsible for three of the selected PHC units. Each researcher visited their assigned units twice weekly throughout the data collection period. Recruitment during each visit continued until the target sample size was achieved.

### Data collection tool

Data were collected between December 2023 and February 2024 using a validated structured questionnaire developed by the researchers after an extensive literature review. The questionnaire underwent content validation by two independent public health experts to ensure clarity, relevance, and appropriateness of items. The questionnaire was filled out during an interview with each mother attending the PHC units after explaining the aim of the study in Arabic. The questionnaire included the following sections:

1. Socio-demographic section, including socioeconomic status assessed using the validated scale developed by El-Gilany et al. [13].
2. Obstetric history and the last delivery experience, including parity, mode and place of last delivery, number of previous CS, doctor’s suggestion of the mode of delivery, and reported medical and non-medical indications for CS. In this study, a medical indication for CS was defined as any obstetric or maternal condition for which CS is clinically recommended to improve maternal or neonatal outcomes, such as fetal distress, abnormal presentation, placenta previa, multiple pregnancy, or maternal health conditions like hypertension or diabetes [14, 15]. Non-medical (elective) indications refer to CS performed without an established clinical necessity, often based on maternal request, convenience, fear of labor pain, or perceived safety considerations [16].The classification of CS indications in this study was based on whether they represented a verifiable clinical diagnosis or a subjective/logistical driver. While ‘Prior cesarean section’ was categorized as a medical cause due to its influence on local practice patterns, we distinguished between ‘Failure of labor progress’ (medical) and ‘Prolonged labor’ (non-medical). Additionally, the study permitted participants to report multiple indications per delivery, acknowledging that the path to a CS is often multifactorial, involving a combination of clinical history, maternal preference, and logistical considerations. The medical causes included failure of labor progress, cephalo-pelvic disproportion, mal-presentation, fetal distress, umbilical cord prolapse, placental abruption, twins, post-date, mother’s medical condition, prior CS, and others. Non-Medical causes included fear of labor, prolonged labor, previous bad experience, long distance to the hospital, and others [2].
3. Preference for next delivery and reasons for choosing CS, including perceived safety for the baby, less pain, better for baby’s health, suitable option for tubal ligation, known time of delivery, husband preference, and others [17]. Multiple answers were allowed.

### Statistical analysis

All collected data were tabulated using the Statistical Package for Social Sciences (SPSS) version 26. No missing data was found. All data were presented as qualitative data in the form of frequencies and percentages. Univariate analysis was performed to determine the risk factors associated with the mode of last delivery and preference for the next delivery. Also, multivariate logistic regression analysis was performed to identify the independent significant predictors. The multivariate logistic regression model was constructed using a purposeful selection process; all variables that demonstrated statistical significance in the bivariate analysis (*p* < 0.05) were entered into the final model. Before entry, multicollinearity was assessed using the Variance Inflation Factor (VIF). All predictors showed VIF values below 3.5, confirming the absence of significant multicollinearity and ensuring the stability of the regression coefficients despite the inclusion of multiple socioeconomic variables. The significance level was set at *p* < 0.05.

### Ethical considerations

Approval of the institutional review board was obtained from the faculty of medicine, Port Said University, [Ethical Review Number (ERN): MED (2/12/2023) s.no (118) FAM_003]. Informed consent was obtained from all participants. The privacy and confidentiality of the participants were respected. The procedures of the study were conducted in agreement with the Declaration of Helsinki. Reporting of the study was done according to STROBE guidelines.

## Results

The study included 179 women, half of them were below 23 years, 65% had university education, 45% were not working, nearly half were multiparous, and 60.3% had their last delivery in private clinics or hospitals. Nearly 70% (68%) of women had CS for their last delivery. The rate of CS was significantly higher among women in the higher age groups (e.g., women > 30 years had CS three times higher than women < 23 years, *p* = 0.01). Also, the rate of CS was higher among women who had higher educational level, working in professional or administrative work, or had higher income (Crude OR (95% CI): 5.7 (2.4–13.2), 3.8 (1.4–10.1), 14.7 (5.3–40.9), *p* value: <0.001, 0.005, < 0.001, respectively). Similarly, women whose husbands had higher educational levels, or working as professionals or in administrative work, had higher CS rates (Crude OR (95% CI): 6.1 (2-18.2), 8.4 (3.6–19.5), respectively, *p* value: <0.001for both) (Table 1).

**Table 1:** Sociodemographic characteristics of married women according to mode of delivery, Port Said City, Egypt, December 2023–February 2024 (*n* = 179)

Total	Total *N* (%)	CS *n* (%)	COR (CI 95%)	*P*-value
	179 (100)	122 (68.1)		
Age (Yrs.)				
< 23	38	19 (50)	1 (r)	
23–26	45	28 (62.2)	1.6(0.7-4)	0.263
27–30	45	36 (80)	4 (1.5–10.5)	0.004^*^
> 30	51	39 (76.5)	3.3 (1.3–8.1)	0.01^*^
Educational level				
Illiterate/Read &write	31	13 (41.9)	1 (r)	
Basic/2ry education	31	15 (48.4)	1.3 (0.5–3.5)	0.61
University/Postgraduate	117	94 (80.3)	5.7 (2.4–13.2)	< 0.001^*^
Occupation				
Housewife	81	51 (63)	1 (r)	
Unskilled/Skilled worker	53	32 (60.4)	0.9 (0.6–1.4)	0.763
Professional/administrative	45	39 (86.7)	3.8 (1.4–10.1)	0.005^*^
Husband education				
Illiterate/Read &write	16	6 (37.5)	1 (r)	
Basic and 2ry education	28	10 (35.7)	0.9 (0.3–3.3)	0.906
University/Postgraduate	135	106(78.5)	6.1 (2-18.2)	< 0.001^*^
Husband occupation				
Unskilled worker	42	16 (38.1)	1 (r)	
Skilled worker	51	34 (66.7)	3.3 (1.9–7.6)	0.006^*^
Professional	86	72 (83.7)	8.4 (3.6–19.5)	< 0.001^*^
Family income				
Not sufficient	32	10 (31.3)	1 (r)	< 0.0001^*^
Sufficient	78	52 (66.7)	4.4 (1.8–10.6)	< 0.001^*^
Able to save	69	60 (87)	14.7 (5.3–40.9)	< 0.001^*^

*CS* Caesarean Section, *COR* crude odds ratio, *CI* Confidence Interval

Fisher’s exact, otherwise the Chi-square test was used

^*^Significant at *p* < 0.05

Caesarean Section rates were higher among women who were multiparous, or had their delivery conducted in a private hospital/clinic (Crude OR (95% CI): 1.9(1.01–3.6), 9 (4.4–18.5), *p*: 0.045, < 0.001, respectively). Women whose attending doctor did not decide on the mode of delivery (during early labor) were 17 times more likely to undergo CS than those whose doctor recommended vaginal delivery (Crude OR (95% CI): 17.4 (5.4–56.1), *p* value: <0.001). (Table 2)

**Table 2:** Obstetric characteristics of married women (*n* = 179) according to mode of delivery, Port Said City, Egypt, December 2023–February 2024

Obstetric characteristics	Total *N* (%)	CS *n* (%)	COR (CI 95%)	*P*-value
Total	179 (100)	122 (68.1)		
Parity				
Primipara	81	49 (60.5)	1 (r)	0.045^*^
Multipara	98	74 (74.5)	1.9(1.01–3.6)	
Place of delivery				
Governmental hospital	71	29 (40.8)	1 (r)	< 0.001^*^
Private hospital/clinic	108	93 (86.1)	9 (4.4–18.5)	
Doctor’s opinion/suggestion				
Vaginal	37	4 (10.8)	1 (r)	
No clear suggestion	59	40 (67.8)	17.4 (5.4–56.1)	< 0.001^*^
CS	83	78 (94)	128 (32.5–509)	< 0.001^*^

*CS* Caesarean Section, *COR* crude odds ratio, *CI* Confidence Interval

^*^Significant at *p*<0.05

**Fig. 1:**
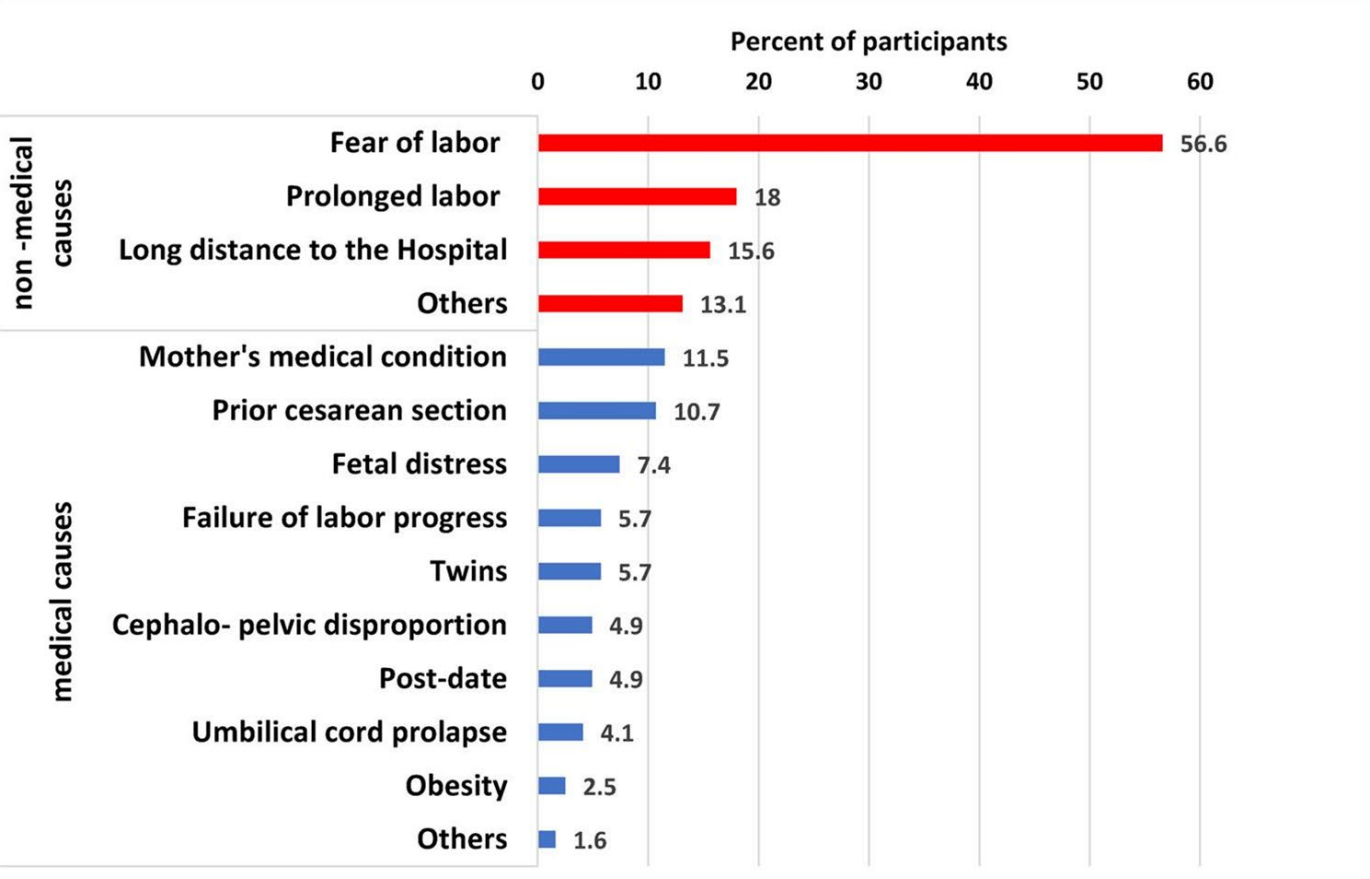
Causes of Caesarean section among married women, Port Said City, Egypt, (*n* = 122), December 2023–February 2024

Results showed that non-medical causes were more prevalent than medical causes, as fear of labor was the most common cause (56%), followed by prolonged labor (18%), and long distance to hospital (15.6%). Regarding medical causes, a mother’s medical condition was prevalent among 11.5% of women, followed by prior CS among 10% of women. Figure (1).

**Fig. 2:**
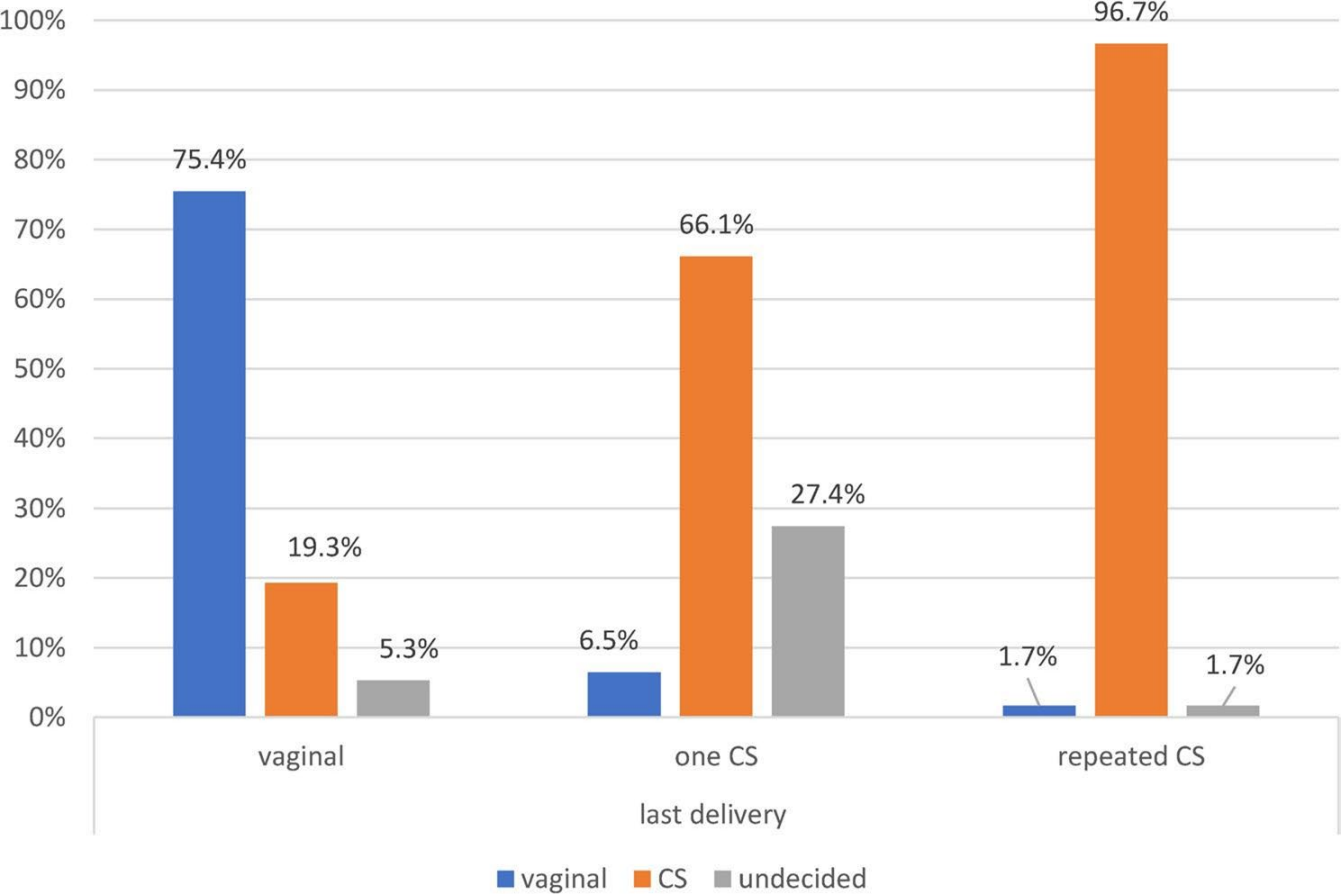
Preference for the mode of next delivery among women by previous delivery, Port Said City, Egypt, December 2023–February 2024

Figure 2 illustrates that preference for CS differed by previous mode of delivery, being 19.3% among women who had a vaginal delivery compared with 66.1% among those whose last delivery was by CS. Overall, 61.5% of women preferred CS for their next delivery.

**Fig. 3:**
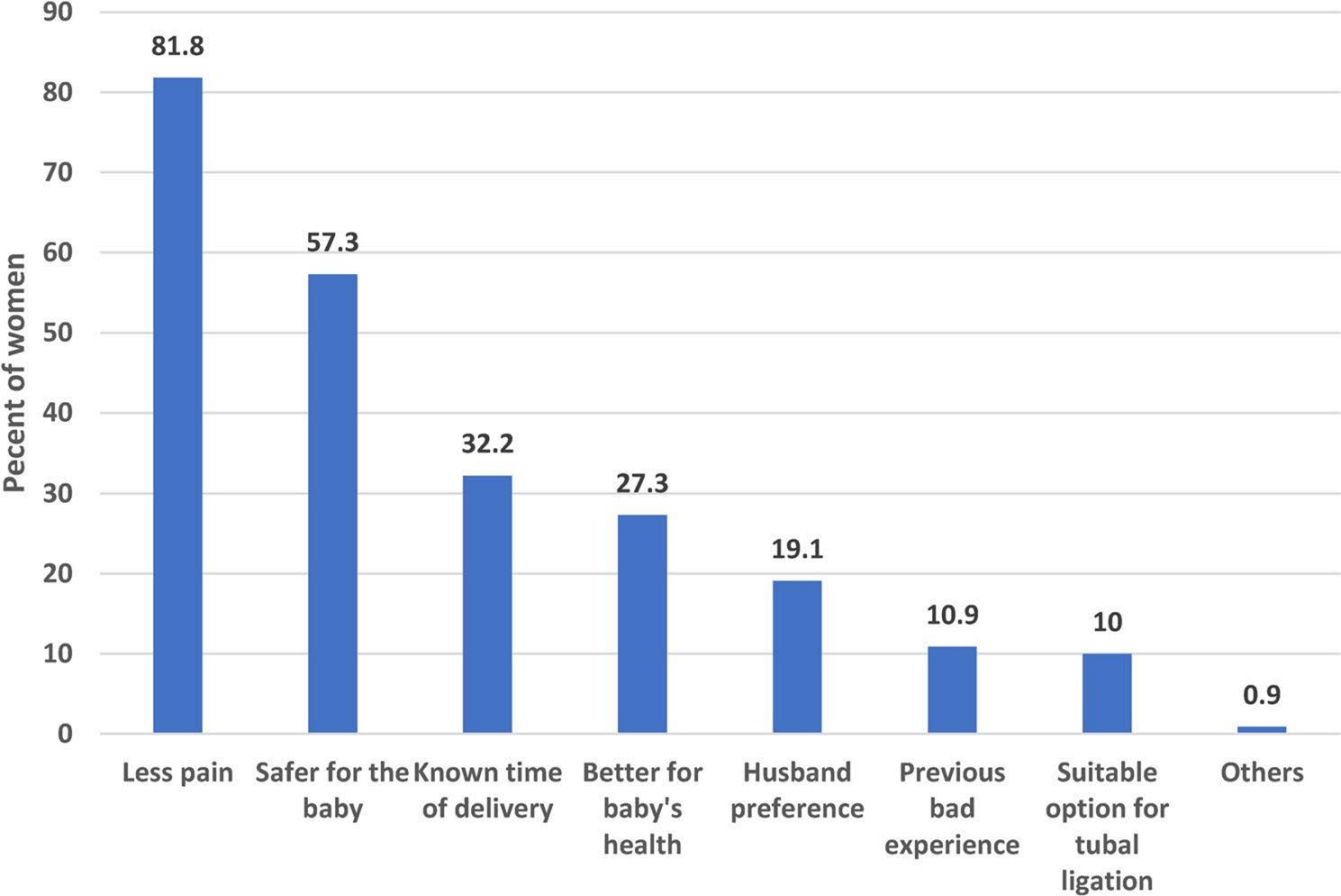
Main reasons for preferring caesarean section for the next delivery (*n* = 110), Port Said City, Egypt, December 2023–February 2024

The most common causes of this preference, as reported by women, were less pain (81.8%), followed by the belief that CS is safer for the baby (57.3%), and the knowledge of the time of delivery (32.2%). Figure (3).

All sociodemographic and obstetric and delivery-related factors associated with the preference of CS among women, shown in Tables (3, 4), were significantly associated with the preference of CS except parity. Having CS for the last delivery significantly increased the likelihood of the CS preference among women (Crude OR (95% CI): 18(8-40.02), *p* value < 0.001). Moreover, having emergency CS for the last delivery was significantly associated with less preference of CS (Crude OR (95% CI): 0.13(0.05–0.35), *p* value < 0.001).

**Table 3:** Preferences for future delivery according to sociodemographic characteristics (*n* = 179)

Total	Total *N*	Prefer CS No. (%)	COR (CI 95%)	*P*-value
	179	110 (61.5)		
Age (Yrs.)				
< 23	38	20 (52.6)	1 (r)	
23–26	45	25 (55.6)	1.2 (0.5–2.7)	0.790
27–30	45	31 (68.9)	2 (0.8–4.9)	0.129
> 30	51	34 (66.7)	1.8 (0.8–4.3)	0.180
Educational level				
Illiterate/Read &write	31	11(35.5)	1 (r)	
Basic/2ry education	31	12(38.7)	1.1 (0.4–3.2)	0.793
University/Postgraduate	117	87(74.4)	5.3 (2.3–12.3)	< 0.001^*^
Occupation				
Housewife	81	45 (52.8)	1 (r)	
Unskilled/Skilled worker	53	28 (61.6)	0.89(0.4–1.8)	0.757
Professional/administrative	45	37 (82.2)	3.7(1.5–8.9)	0.003
Husband education				
Illiterate/Read &write	16	1(6.3)	1 (r)	
Basic and 2ry education	28	9(32.1)	7.1 (0.8–62.5)	0.049^#*^
University/Postgraduate	135	100(74.1)	42.9 (5.5-336.4)	< 0.001^*^
Husband occupation				
Unskilled worker	42	10(23.8)	1 (r)	
Skilled worker	51	35(68.6)	7 (2.8–17.6)	< 0.001^*^
Professional	86	65(75.6)	9.9 (4.2–23.5)	< 0.001^*^
Family income				
Not sufficient	32	6(18.8)	1 (r)	
Sufficient	78	53(67.9)	9.2 (3.4–25.1)	< 0.001^*^
Able to save	69	51(73.9)	12.3 (4.4–34.7)	< 0.001^*^
Repeated	60	58(96.7)	14.85(3.3–66.9)	

*CS* Caesarean Section, *COR* crude odds ratio, *CI* Confidence Interval

^*^Significant at *p* < 0.05

^#^Fisher’s exact, otherwise the Chi square test was used

**Table 4:** Preferences for future delivery according to obstetric and delivery-related factors (*n* = 179)

Obstetric and Delivery-Related Factors	Total *N*	Prefer CS No (%)	COR (CI 95%)	*P*-value
Total	179	110 (61.5)		
Parity				
Primigravida	81	46(56.8)	1 (r)	0.281
Multipara	98	64(65.3)	1.4(0.78–2.62)	
Place of delivery				
Governmental hospital	71	24(33.8)	1 (r)	< 0.001^*^
Private hospital/clinic	108	86 (97.6)	7.7 (3.9–15.1)	
Mode of last delivery				
Vaginal	57	11(19.3)	1 (r)	< 0.001^*^
CS	122	99(81.1)	18(8-40.02)	
Type of CS (*n* = 122)				
Elective	79	73(92.4)	1 (r)	< 0.001^*^
Emergency	43	26(60.5)	0.13(0.05–0.35)	
Numbers of CS (*n* = 122)				
First CS	62	41(66.1)	1 (r)	< 0.0001^*^
Repeated	60	58(96.7)	14.85(3.3–66.9)	

*CS* Caesarean Section, *COR* crude odds ratio, *CI* Confidence Interval

^*^Significant at *p*<0.05

Table 5 shows the results of multiple logistic regression of the predictors of the mode of delivery and the preference for the next delivery. The independent significant predictors of CS delivery among women were higher income and the doctor’s opinion/suggestion. Women with sufficient income had CS ten times more than women with insufficient income [Adjusted odds ratio (AOR) (95% Confidence Interval (CI) 10 (2.7–36.9), *p* value < 0.001). Also, women whose doctors did not suggest a mode of delivery late in pregnancy during planning for delivery had CS nearly 20 times more than women whose doctors suggested vaginal delivery [AOR (95% CI): 19.2 (5.5–67.1), *p* value < 0.001]. The independent significant predictors of CS preference were the husband’s education and the mode of last delivery. Higher husband education and having a previous CS increased the likelihood of preferring CS for the next delivery [AOR (95% CI): 12.082 (1.2-124.3), 14.7 (6.2–34.6), *p* value: 0.036, < 0.001, respectively].

**Table 5:** Regression analysis of predictors of the mode of last delivery and preference for the mode of next delivery among women, Port Said City, Egypt, December 2023–February 2024

Predictors	B	*P*	AOR (95% CI)	
Mode of last delivery				
Income				
not sufficient	-		1(r)	Constant: -4.341 Model χ2: 110.395, *p* < 0.001^*^, % correctly predicted:85.5%
sufficient	2.303	< 0.001^*^	10 (2.7–36.9)	
able to save	3.180	< 0.001^*^	24 (5.6-103.2)	
Doctor’s opinion/ suggestion				
vaginal	-		1(r)	
CS	5.259	< 0.001^*^	192 (39.5-936.7)	
no clear suggestion	2.954	< 0.001^*^	19.2 (5.5–67.1)	
Preference for the next delivery mode				
Husband education				
illiterate/read & write	-		1(r)	Constant: -4.443 Model χ2: 85.79, % correctly predicted: 83.2%, *p* < 0.001^*^
basic and secondary	2.492	0.036	12.082 (1.2-124.3)	
university and post	3.587	< 0.001^*^	36.1 (4.2-312.4)	
Mode of last delivery				
vaginal	-		1(r)	
CS	2.687	< 0.001^*^	14.7 (6.2–34.6)	

*AOR* Adjusted odds ratio

^*^Significant at *p* < 0.05

## Discussion

Caesarean Section rates have been rising globally, associated with factors like maternal age, education level, and socioeconomic status. According to the WHO, the ideal CS rate should not exceed 10–15% in any population [18]. However, many countries report rates above this threshold, raising concerns about the appropriateness of surgical deliveries [19]. Understanding the demographic and socioeconomic influences on CS rates is crucial for developing strategies to optimize maternal care [20]. This study investigates factors associated with CS rates and preferences among women in a specific demographic, describing patterns and correlates of these trends.

In the present study, more than two-thirds of participants were delivered by CS, reflecting a pattern similar to that reported in other Eastern Mediterranean countries. While obstetric indications such as fetal distress and previous CS were commonly cited, non-obstetric reasons, including fear of labor and scheduling preferences, emerged as prominent influences, underscoring the role of perceptions and health system practices alongside clinical need.

The high prevalence of C-sections observed in this study (68%) must be interpreted within the unique obstetric landscape of Port Said governorate. According to the 2021 Egypt Family Health Survey (EFHS), Port Said governorate recorded the highest CS rate in the country at 91.3%. Our finding of 68% is notably lower than the regional average, which may be attributed to our sampling at Primary Health Care (PHC) units. PHC attendees often represent lower-to-middle socioeconomic strata, whereas the highest CS rates in Egypt are concentrated in the private sector. While the use of convenience sampling is a limitation, the inclusion of women attending compulsory vaccination clinics provided a relatively diverse cross-section of the population. Consequently, the rates reported here likely represent a conservative estimate of the broader trend in Port Said, reflecting a sub-population with slightly higher utilization of public-sector care compared to the general governorate population [21].

In some cultures, CS is seen as safer than or more modern than vaginal delivery, and hospital protocols, staffing, or resources may be associated with higher CS rates. A study conducted by Abdel-Tawab et al. (2018) concluded that the sharp increase in the prevalence of CS deliveries in Egypt, with a CS rate of 52%, suggests overuse or inappropriate indications. Factors associated with this include financial incentives, doctors’ desire for better time control, fear of medical litigation, unclear medical protocols, and limited practice opportunities for junior doctors, a shortage of pain relief drugs in public hospitals, and a lack of trained anaesthesiologists for epidural anaesthesia [22].

Moreover, a study by Wahdan et al. found that the CS rate in six Egyptian governorates was 55.1% over the three years preceding the study, with the highest rate at 67.8% in Behira and the lowest at 49.0% in Assiut. The CS rate was higher in rural areas compared to urban areas, though the difference was not statistically significant. High CS rates were significantly associated with higher social class and having fewer children (≤ 3) [23].

Recent evidence from Türkye further supports the regional pattern of elevated CS rates observed in the Eastern Mediterranean. In a large cross-sectional study conducted at a Turkish tertiary care center involving 68,944 deliveries, the overall CS rate was 55.0%, with 56.2% of cases attributable to previous uterine surgery (including prior CS) and 24.3% due to fetal distress, while other fetal-related indications accounted for smaller proportions (multiple gestation 6.2%, malpresentation 4.2%, macrosomia 1.9%) [24].

Similarly, an analysis using the Robson Ten Group Classification in 63,809 deliveries found an overall CS rate of 54.2%, increasing from 42.2% in 2020 to 59.9% in 2024, and identified that Group 5 (previous CS) related to 23.9% of all CSs. These figures mirror the elevated CS prevalence in the current study and reinforce the influence of prior CS history, clinical practice patterns, and systemic factors in driving high surgical delivery rates across Eastern Mediterranean health systems [25].

Betran et al. analyzed global and regional trends in CS rates using data from 2010-2018, covering 154 countries and 94.5% of world live births. They found that 21.1% of women worldwide gave birth by CS, with regional averages ranging from 5% in sub-Saharan Africa to 42.8% in Latin America and the Caribbean. Since 1990, CS rates have increased in all regions, with the most significant rises in Eastern Asia (44.9%), Western Asia (34.7%), and Northern Africa (31.5%). Projections indicate that by 2030, 28.5% of women globally would give birth by CS, with rates expected to range from 7.1% in sub-Saharan Africa to 63.4% in Eastern Asia [19].

The results of this study highlight significant associations between demographic and socioeconomic factors and rates and preferences for CS among women. The findings reveal critical insights into the influences of age, education, and income on the likelihood of undergoing a CS and the preference for future deliveries. Consistent with prior literature, CS rates were higher among women aged over 30 years, reflecting both increased medical risk and provider perceptions of advanced maternal age [26, 27]. This aligns with other studies [28–30], which found similar patterns in their analysis of delivery modes across age groups. Higher educational attainment and professional employment were also associated with CS, likely reflecting greater access to services, stronger negotiation power, and preference for perceived convenience or safety [31–33]. Other studies have shown that educated women are more informed about their choices and may prefer CS for perceived benefits, such as scheduling convenience or less pain during labor [34, 35]. Our findings extend this evidence by showing that these demographic factors also shape future delivery preferences, not only past delivery outcomes.

Our findings reveal that multiparous women and those who delivered in private clinics/hospitals had higher rates of CS. Previous research supports this, indicating that multiparous women might experience different expectations and healthcare provider biases toward CS [36–38]. The preference for private institutions is often linked to better perceived quality of care and resources, which can influence the decision-making process regarding delivery methods [39]. A study by Mobarak & Sultan [40] further emphasized the role of healthcare settings in shaping delivery options, noting that women in private care often receive different counseling than those in public systems. On the other hand, Ivie, et al. concluded that the prevalence of CS was higher among primigravida women compared to multiparous women [41].

The prominence of non-obstetric reasons especially fear of labor highlights a critical gap in antenatal education and counseling. Psychological factors and misconceptions about childbirth safety have been widely reported [42, 43]; however, this study adds value by demonstrating their direct association with both CS uptake and future delivery preferences in a real-world high-prevalence setting.

Previous CS strongly predicted preference for repeat CS, confirming the lasting influence of birth experiences on future decisions [44, 45]. Notably, the strong association between doctors’ recommendations and CS use observed in this study illustrates the central role of provider-patient communication, particularly where delivery plans are not discussed early. This finding highlights a modifiable system-level factor that may be related to unnecessary CS use [46–53].

The strong association observed in the current study between the absence of a clear physician recommendation and CS preference highlights the pivotal role clinicians play in shaping women’s decisions about the mode of delivery. Studies have shown that providers’ recommendations and the level of confidence women place in their birth practitioner are significantly associated with a higher likelihood of choosing cesarean delivery, underscoring the influence of physician guidance on maternal preference for CS even in non-medical contexts [17, 54]. However, it remains unclear whether this association reflects late decision-making by women, defensive practice by physicians, or systemic issues in antenatal counseling. The present study design cannot distinguish among these possibilities, and further research is needed to explore the timing of decision-making, healthcare provider practices, and structural barriers that may influence CS preference. Promoting shared decision-making and providing evidence-based physician guidance could help reduce unnecessary cesarean sections and support more informed maternal choices.

PHC-based interventions, such as structured antenatal education and counseling, can help address fear of labor and misconceptions about cesarean section, supporting more informed delivery decisions and potentially reducing unnecessary CS. Strengthening provider–patient communication during routine antenatal visits has been shown to improve maternal confidence and promote safer childbirth choices [55].

In summary, this study advances existing literature by demonstrating that high CS rates are driven not only by demographic and obstetric factors but also by non-obstetric concerns, prior experiences, and provider influence. These findings emphasize the importance of addressing psychosocial factors and strengthening shared decision-making to reduce unnecessary CS use in high-prevalence settings.

### Strengths and limitations

This study provides a comprehensive assessment of CS rates and women’s preferences, incorporating socio-demographic, economic, and psychosocial factors. However, the study also has notable limitations. Its cross-sectional design precludes causal inference, and the data were self-reported, which may introduce recall or reporting bias. Participants were recruited only from PHC units (primarily lower-to-middle socioeconomic strata), limiting generalizability to the broader population and providing a conservative estimation of CS rates. Additionally, information on nationality, migrant status, or refugee status was not collected. This is important because migrant or refugee women may experience different patterns of CS and face distinctive obstacles to gaining access to care, which were not captured in the study analysis. Some indications exist in a clinical “gray zone.” The lack of standardized clinical audits or partogram data means these classifications rely on maternal perception of the provider’s decision.

## Conclusion

This study identifies key factors influencing CS rates and delivery preferences. Caesarean Section was more common among older, more educated, and higher-income women, with non-obstetric reasons such as fear of labor playing a prominent role. Prior CS, doctors’ recommendations, and husbands’ education significantly shaped women’s preferences for future deliveries. These findings highlight the need for targeted interventions that provide balanced information and address misconceptions about CS to support informed decision-making.

Based on the study findings, improving antenatal education is essential to address non-obstetric drivers of CS, particularly fear of labor and misconceptions about delivery safety. Clear, evidence-based counseling, especially for women with previous CS and strengthened provider-patient communication, can support informed, shared decision-making. These measures may help align delivery choices more closely with clinical need and women’s preferences.

## Supplementary Information

Supplementary Material 1.

## Data Availability

All data generated or analyzed during this study are included in this article. In addition, the related datasets are available from the corresponding author upon reasonable request.

## Abbreviations

AOR: Adjusted Odds Ratio
CAPMAS: Central Agency for Public Mobilization and Statistics
CDMR: Caesarean Delivery on Maternal Request
CI: Confidence Interval
COR: Crude Odds Ratio
CS: Caesarean Section
MOHP: Ministry of Health and Population
OR: Odds Ratio
PHC: Primary Health Care
SPSS: Statistical Package for the Social Sciences
STROBE: Strengthening the Reporting of Observational Studies in Epidemiology
WHO: World Health Organization
