# Supplementary material for: Prevalence, indications, and preference of caesarean section deliveries among women attending primary health care units in Port-Said City, Egypt
Source: J Egypt Public Health Assoc. 2026 May 11;101:15. doi: 10.1186/s42506-026-00218-1 (PMC13161398; doi:10.1186/s42506-026-00218-1)
Supplement: Supplementary file 1 — Supplementary Material 1. [file 42506_2026_218_MOESM1_ESM.docx]

**Appendix (1)**

**Prevalence, Indications and preference of Caesarean sections deliveries among women attending primary health care units in Port-Said city**

| 1. **Sociodemographic data** | |  |
| --- | --- | --- |
| **Health care site:** | |  |
| ***Name****:* | |  |
| ***Age****:* | |  |
| ***Level of education****: Illiterate Read & write*  *Primary/ preparatory education secondary education*  *University/ Postgraduate degree* | |  |
| ***Occupation****: Housewife*  *Skilled worker*  *Unskilled worker*  *Professional* | |  |
| ***Husband education****: Illiterate Read & write*  *Primary/ preparatory education secondary education*  *University/ Postgraduate degree* | |  |
| ***Husband Occupation****:*  *Not working  Skilled worker*  *Unskilled worker*  *Professional/Administrative* | |  |
| ***Income****:* *Not sufficient for routine expenses*  *Not sufficient for emergency Sufficient for routine expenses*  *Able to save* | |  |
| 1. **Obstetric history** | |  |
| Parity | *Primipara*  *Multipara* | |
| Mode of last delivery | *Vaginal*  *C.S.* | |
| Place of delivery | *Governmental hospital*  *Private hospital*  *Private clinic* | |
| Number of C.S. | *First C.S.*  *Repeated* | |
| Type of C.S. | *Elective*  *Emergency* | |
| Doctor's suggestion during planning for delivery | *Vaginal*  *C.S.*  *No clear suggestion* | |
| The cause of C.S. | *Medical*  *Non-medical* | |
| ***Medical Causes*** | | |
| *Failure of labor progress* | | |
| *Cephalopelvic disproportion (Macrosomia , Hydrocephalus ,..)* | | |
| *Malpresentation (Breech ,..)* | | |
| *Fetal distress ( Heart condition, Oligohydramnios, …)* | | |
| *Umbilical cord prolapse* | | |
| *Placental abruption* | | |
| *Twins* | | |
| *Prior cesarean section* | | |
| *Post-date* | | |
| *Obesity* | | |
| *Mother's medical condition (HTN, DM ,...)* | | |
| *Others………………………………………………………………………………………* | | |
| ***Non medical causes*** | | |
| *Fear of labor pain* | | |
| *Prolonged labor pain* | | |
| *Long distance to hospital* | | |
| *Others: ……………………………………………….* | | |

| ***Preference for next delivery****: Vaginal C.S. Undecided* |
| --- |
| ***If choosing C.S: Reasons for choosing C.S.*** |
| *Safer for the baby* |
| *Less pain* |
| *Better for baby's health* |
| *Suitable option for tubal ligation* |
| *Known time of delivery* |
| *Previous bad experience* |
| *Husband preference* |
| *Others* |
